# Liprin-α1 Expression in Tumor-Infiltrating Lymphocytes Associates with Improved Survival in Patients with HPV-Positive Oropharyngeal Squamous Cell Carcinoma

**DOI:** 10.1007/s12105-023-01565-7

**Published:** 2023-06-19

**Authors:** Anni Sjöblom, Henna Pehkonen, Lauri Jouhi, Outi Monni, Reija Randén-Brady, Piia-Riitta Karhemo, Jussi Tarkkanen, Caj Haglund, Petri Mattila, Antti Mäkitie, Jaana Hagström, Timo Carpén

**Affiliations:** 1 Department of Pathology, University of Helsinki and Helsinki University Hospital, PO Box 21, 00014 Helsinki, Finland; 2 Applied Tumor Genomics Research Program, Faculty of Medicine, University of Helsinki, Helsinki, Finland; 3 Department of Otorhinolaryngology, Head and Neck Surgery, University of Helsinki and Helsinki University Hospital, Helsinki, Finland; 4 Applied Tumor Genomics Research Program and Department of Oncology, Clinicum, Faculty of Medicine, University of Helsinki, Helsinki, Finland; 5 Research Programs Unit, Translational Cancer Medicine and Department of Surgery, University of Helsinki and Helsinki University Hospital, Helsinki, Finland; 6 Division of Ear, Nose and Throat Diseases, Department of Clinical Sciences, Intervention and Technology, Karolinska Institutet and Karolinska Hospital, Stockholm, Sweden; 7 Department of Pathology and Research Programs Unit, Translational Cancer Medicine, University of Helsinki, Helsinki, Finland; 8 Department of Oral Pathology and Oral Radiology, University of Turku, Turku, Finland; 9 Departments of Pathology and of Otorhinolaryngology, Head and Neck Surgery and Research Program in Systems Oncology, Faculty of Medicine, University of Helsinki, Helsinki, Finland

**Keywords:** OPSCC, HPV, Prognosis, Biomarker

## Abstract

**Background:**

Liprin-α1 is a scaffold protein involved in cell adhesion, motility, and invasion in malignancies. Liprin-α1 inhibits the expression of metastatic suppressor CD82 in cancers such as oral carcinoma, and the expression of these proteins has been known to correlate negatively. The role of these proteins has not been previously studied in human papillomavirus (HPV)-related head and neck cancers. Our aim was to assess the clinical and prognostic role of liprin-α1 and CD82 in HPV-positive oropharyngeal squamous cell carcinoma (OPSCC) in comparison to HPV-negative OPSCC.

**Methods:**

The data included 139 OPSCC patients treated at the Helsinki University Hospital (HUS) during 2012–2016. Immunohistochemistry was utilized in HPV determination and in biomarker assays. Overall survival (OS) was used in the survival analysis.

**Results:**

Stronger expression of liprin-α1 in tumor-infiltrating lymphocytes (TILs) was linked to lower cancer stage (*p* < 0.001) and HPV positivity (*p* < 0.001). Additionally, we found an association between elevated expression of liprin-α1 and weak expression of CD82 in tumor cells (*p* = 0.029). In survival analysis, we found significant correlation between favorable OS and stronger expression of liprin-α1 in TILs among the whole patient cohort (*p* < 0.001) and among HPV-positive patients (*p* = 0.042).

**Conclusions:**

Increased liprin-α1 expression in the TILs is associated with favorable prognosis in OPSCC, especially among HPV-positive patients.

**Supplementary Information:**

The online version contains supplementary material available at 10.1007/s12105-023-01565-7.

## Introduction

Oropharyngeal squamous cell carcinoma (OPSCC) is a form of head and neck malignancy that has been associated with adverse prognosis (5-year survival 50–80%) [1]. Currently, the majority of newly diagnosed OPSCC patients carry human papillomavirus (HPV) [2], and the characteristics of HPV-positive OPSCC differ clearly from the HPV-negative form of the disease [3]. Thus, HPV-positive OPSCC has been established as a separate disease entity [4]. In addition to HPV, smoking and heavy alcohol use are patient characteristics known to increase the risk of OPSCC as well as to affect patient survival [5]. An ongoing discussion on whether the treatment of HPV-positive OPSCC should be de-escalated continues, but to date, there remain no conclusive findings resulting in de-escalation recommendations [6]. Further information on the role of HPV in treatment response and tumor pathogenesis is desired.

The incidence of HPV-positive OPSCC in particular has been rising rapidly in the Western world, partly because of the spread of HPV [7]. Despite the improved survival of HPV-positive OPSCC [5], HPV-negative OPSCC remains a burden on healthcare, as well as on the quality of patient’s life, due to complicating factors such as common recurrences and adverse treatment responses [5]. Thus, to improve outcomes, advocating for the customization of treatment modalities such as immunotherapies is crucial, and polishing diagnostic methods is desirable. Incorporating biomarkers in the management of other malignancies has been known to facilitate the development of individualized, targeted treatments [8]. Similarly, biomarkers could provide potential benefits in the management of OPSCC. Biomarker assays include various benefits, most significantly the prospects of monitoring the disease as well as assessing survival and treatment responses [9]. However, the introduction of biomarkers into clinical use is challenging [10]. To date, aside from HPV/p16 determinations [11], biomarker assays have not been used in the selection of treatment modalities among OPSCC patients.

The objective of our study was to assess the role of two tissue-specific biomarkers known as liprin-α1 and CD82 in OPSCC. Both markers are proteins known to have separate functions in cellular motility and adhesion [12, 13]. Liprin-α1 has been observed to possess cancer-promoting abilities, and elevated expression has been linked with impaired prognosis in head and neck cancers, as well as in breast cancers [14–17]. In contrast, previous studies have shown that CD82 is a tumor suppressor [18], and low CD82 expression has been associated with increased probability of metastatic disease [19]. It has further been discovered that liprin-α1 inhibits CD82 in cancers of the breast and of the head and neck region [16]. To assess the potential of these biomarkers in the management of HPV-positive and HPV-negative OPSCC, we compared the expression of liprin-α1 and CD82 in tumor cells to clinical characteristics and survival of the patients. Additionally, the expression of these biomarkers in tumor-infiltrating inflammatory cells was included in the analysis, as they are considered a significant prognostic factor in head and neck cancers [20].

## Materials and Methods

### Study Cohort and Patient Material

Our study population included 139 newly diagnosed OPSCC patients treated at the Helsinki University Hospital (HUS, Helsinki, Finland) during 2012–2016. Treatment modalities included definitive radiotherapy with or without chemotherapy, or surgery with or without postoperative (chemo)radiotherapy. The inclusion criteria for the study were existing tissue microarray (TMA) slides with adequate tissue material prepared in advance, as well as available HPV status.

Follow-up information and details on clinical characteristics were acquired manually from electronic patient records from the same database as was used in our previous reports [21, 22]. The clinicopathological characteristics included mean age at diagnosis, gender, smoking habit, alcohol use, TNM class (8th edition of American Joint Committee on Cancer staging), stage, grade of differentiation, and tumor site. Clinical characteristics were compared to biomarker assays performed with tumor samples. To further investigate the differences between HPV-positive and HPV-negative OPSCC, we performed additional, corresponding analyses within patient subgroups according to HPV status. Follow-up time was determined as the time period from the date of the end of treatment until the last follow-up date or death.

The study design was approved by the Research Ethics Board at the HUS (Dnr: 51/13/03/02/2013), and institutional study permission was granted. Patients gave written consent prior to participation in the study.

### Tissue Microarrays

TMAs were prepared in advance from formalin-fixed and paraffin-embedded primary tumors for the immunohistochemical (IHC) analysis. Representative areas were selected from hematoxylin and eosin-stained slides, and six core biopsies (one mm in diameter) were detached from each tumor with the assistance of digital software by Auria Biobank (Turku, Finland). The core biopsies were then placed in a separate paraffin block with a tissue microarrayer (Beecher Instruments, Silver Spring, MD, USA). The method is the same as that described previously in our earlier reports [22]. In a few cases, the TMA spots no longer contained tumor tissue, in which case the result was excluded from the IHC analysis.

### HPV Determination by mRNA in situ Hybridization

In situ hybridization (ISH) for high-risk HPV E6/E7 mRNA was carried out with the RNAscope^®^ 2.5 HD Reagent kit (Advanced Cell Diagnostics, Inc., Hayward, CA) for genotypes 16, 18, 26, 31, 33, 35, 39, 45, 51, 52, 53, 56, 58, 59, 66, 68, 73, and 82. An endogenous housekeeping gene HS-PPIB (RNAscope^®^) probe and a bacterial gene DapB, diaminopimelate (RNAscope^®^) probe were used as positive and negative controls, respectively. The methodology is described in detail in the earlier work of Randén-Brady et al., where it was further shown that mRNA ISH is the recommended method for HPV determination in OPSCC [22].

### Biomarker Immunohistochemistry

The primary antibodies used in our analysis were rabbit polyclonal liprin-α1 (Proteintech, Manchester, U.K.) and mouse monoclonal CD82 (Abcam, Cambridge, U.K.). The secondary antibodies used were horseradish peroxidase (HRP) conjugate goat anti-mouse IgG (H + L) (Life Technologies, Rockford, IL, U.S.), and HRP conjugate goat anti-rabbit IgG (H + L) (Life Technologies, Rockford, IL, U.S). The protocol for immunohistochemical staining analyses in our institution is described in detail in our earlier publication [21]. A positive control was applied in both liprin-α1 and CD82 analyses.

**Fig. 1:**
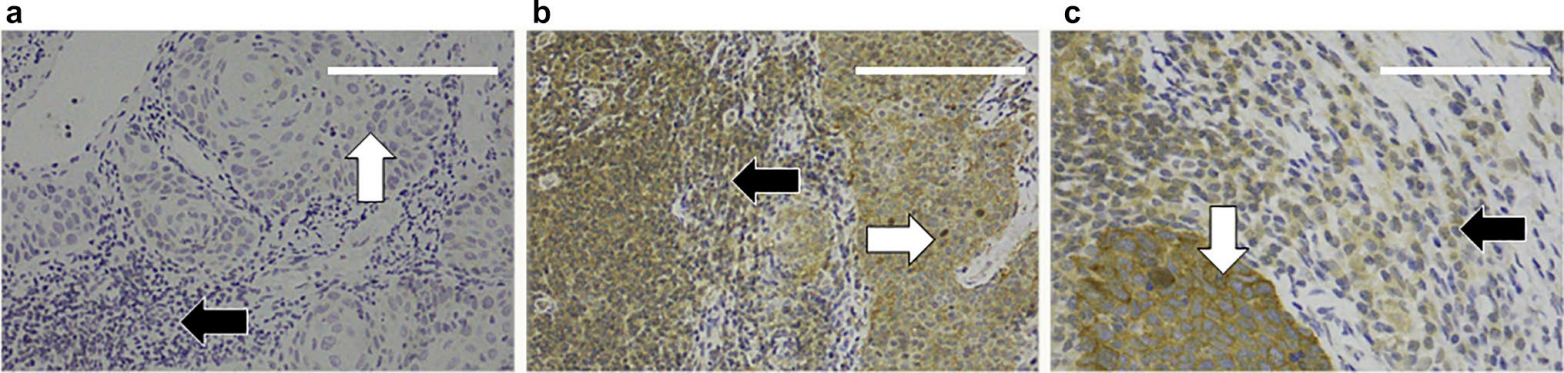
Liprin-α1 immunostaining (**a**) Negative immunostaining in tumor cells (white arrow) and in tumor-infiltrating lymphocytes (TILs) (black arrow), scale bar length 150 µm, magnification × 200 (**b**) Positive immunostaining in tumor cells (white arrow) and in TILs (black arrow), scale bar length 150 µm, magnification × 200 (**c**) Positive immunostaining in tumor cells (white arrow) and in TILs (black arrow), scale bar length 75 µm, magnification × 400

**Fig. 2:**
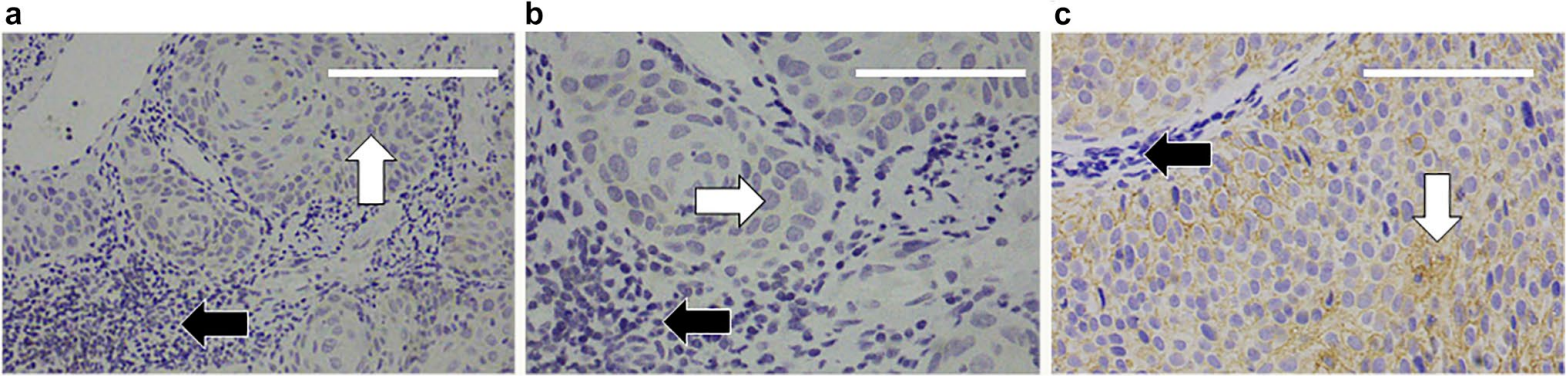
CD82 immunostaining (**a**) Negative immunostaining in tumor cells (white arrow) and in TILs (black arrow), scale bar length 150 µm, magnification × 200 (**b**) Negative immunostaining in tumor cells (white arrow) and in TILs (black arrow), scale bar length 75 µm, magnification × 400 (**c**) Negative immunostaining in TILs (black arrow) and positive immunostaining in tumor cells (white arrow), scale bar length 75 µm, magnification × 400

**Fig. 3:**
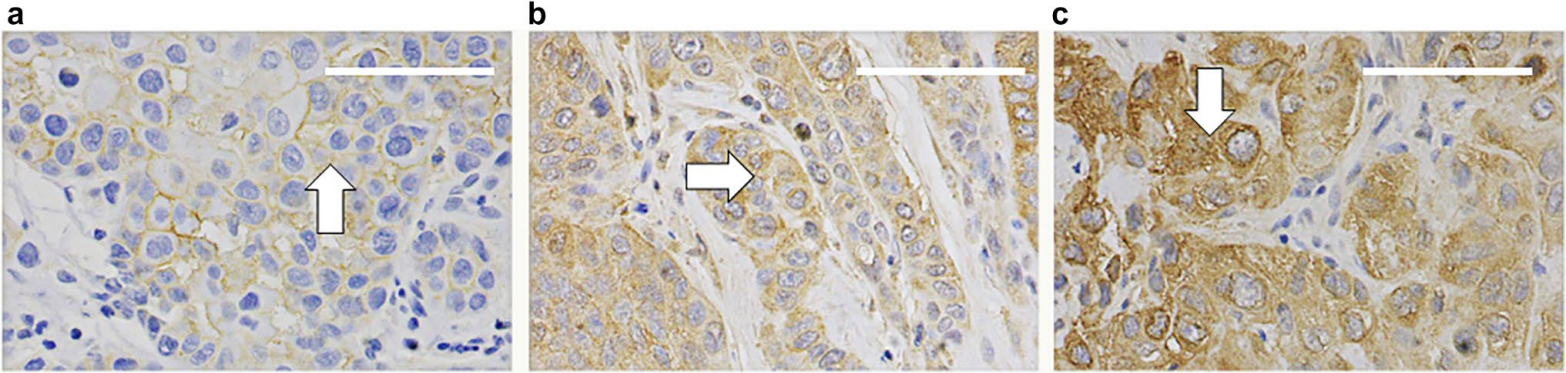
Grading of immunostaining intensity (**a**) weak/light staining (**b**) moderate/medium staining (**c**) strong/dark staining of biomarker, scale bar length 75 µm, magnification × 400

The samples were immunoscored separately by two researchers (J. H. and A. S.). If there was disagreement between the investigators, the consensus was achieved unanimously. The expression of liprin-α1 and CD82 was scored in the tumor cells and in the tumor-infiltrating lymphocytes (TILs). In our samples, the tumor cells can be differentiated from TILs by their nuclei; in tumor cells, the nuclei are large, and their morphology can be diverse, while in TILs, the nuclei are small and uniform in morphology, as illustrated in Figs. 1, 2. The staining of both biomarkers was mainly cytoplasmic. Liprin-α1 expression was scored in tumor cells and in TILs, whereas CD82 expression was scored solely in tumor cells as TILs were consistently negative. As the majority of the samples showed positive immunostaining, the samples were graded according to intensity/darkness of staining (0 = negative/no staining, 1 = weak/light staining, 2 = moderate/medium staining, 3 = strong/dark staining), as presented in Fig. 3.

### Statistical Methods

Statistical analyses were performed separately by three researchers (A.S., T.C., and L.J.), and the results were then compared for validation. We used IBM SPSS software version 27.0 for the statistical analyses. The χ^2^-test and Fischer’s exact test were used to achieve the crosstab comparisons between biomarker expression and clinical characteristics. The independent samples *t* test was used with normally distributed continuous variables. For non-normally distributed continuous variables, the Mann–Whitney test was performed. Overall survival (OS) was utilized to assess survival, determined as the time period between the date of diagnosis and either the end of the follow-up or death of any cause. Log-rank test and Cox proportional hazards test were used for the statistical survival analysis, and the survival curves were illustrated by the Kaplan–Meier method.

To further measure the independent prognostic value of the biomarkers, we first performed univariable Cox test with each variable, with variables receiving *p* values below 0.01 being admitted to the multivariable analysis.

## Results

### Clinical Characteristics

Moderate or strong expression of liprin-α1 in TILs was detected in 81 patients (59.6%) among the whole cohort, and the stronger expression correlated with male gender (*p* = 0.013), lower N class (*p* = 0.004), lower stage (*p* < 0.001), and higher grade of differentiation (*p* = 0.048). We further observed a correlation with smoking and negative or weak expression of liprin-α1 in TILs (*p* = 0.010), as most of non- and ex-smokers (63.2% and 75.6%, respectively) presented stronger immunostaining, whereas most of current smokers (54.4%) presented negative or weak immunostaining. We observed no correlation between liprin-α1 expressions in TILs and in tumor cells. Further, liprin-α1 expression in TILs did not correlate with CD82 expression in tumor cells (Table 1).

**Table 1:** Clinical characteristics according to liprin-α1-expression in tumor-infiltrating lymphocytes

	liprin-α1 0–1	%	liprin-α1 2–3	%	*p* value	Missing / % (*N* = 136)
Number of patients	55	40.4	81	59.6		
Mean age at diagnosis	62.5		60.8		0.289	
Gender					**0.013**	
Male	37	34.9	69	65.1		
Female	18	60.0	12	40.0		
Smoking habit					**0.010**	
Non	14	36.8	24	63.2		
Ex	10	24.4	31	75.6		
Current	31	54.4	26	45.6		
Heavy alcohol use					0.056	23 / 16.9
Non	23	35.9	41	64.1		
Ex	11	68.8	5	31.2		
Current	13	39.4	20	60.6		
T class					0.667	
T1–T2 56	34	39.1	53	60.9		
T3–T4	21	42.9	28	57.1		
N class					**0.004**	
N0–N1 34 34	35	33.7	69	66.3		
N2–N3	20	62.5	12	37.5		
Stage					**< 0.001**	
I–II	28	30.4	64	69.6		
III–IV	27	61.4	17	38.6		
Grade of differentiation					**0.033**	
I	2	66.7	1	33.3		
II	15	60.0	10	40.0		
III	38	35.2	70	64.8		
Tumor site					0.068	
Tonsil	27	32.5	56	67.5		
Base of tongue	16	47.1	18	52.9		
Soft palate	9	64.3	5	35.7		
Posterior wall of oropharynx	3	60.0	2	40.0		
HPV status					**< 0.001**	
HPV +	32	31.7	69	68.3		
HPV −	23	65.7	12	34.3		

*HPV* Human papillomavirus, *liprin-α1 0–1* negative-weak positivity, *liprin-α1 2–3* moderate-strong positivity

***p* < 0.05**

There was moderate or strong expression of liprin-α1 in tumor cells in 104 patients (75.9%) among the whole cohort. We found significant correlation between liprin-α1 and CD82 expression in tumor cells (*p* = 0.029). Among the patients with negative or weak CD82 immunostaining, 71.2% showed moderate or strong immunostaining of liprin-α1. No further correlation was seen between liprin-α1 expression in tumor cells and clinical characteristics (Table 2).

**Table 2:** Clinical characteristics according to liprin-α1-expression in tumor cells

	liprin-α1 0–1	%	liprin-α1 2–3	%	*p* value	Missing / % (*N* = 137)
Number of patients	33	24.1	104	75.9		
Mean age at diagnosis	61.4		61.6		0.886	
Gender					0.913	
Male	26	24.3	81	75.7		
Female	7	23.3	23	76.7		
Smoking habit					0.267	
Non	13	33.3	26	66.7		
Ex	9	22.0	32	78.0		
Current	11	19.3	46	80.7		
Heavy alcohol use					0.513	23 / 16.8
Non	21	32.3	44	67.7		
Ex	3	18.8	13	81.2		
Current	8	24.2	25	75.8		
T class					0.417	
T1–T2 56	19	21.8	68	78.2		
T3–T4	14	28.0	36	72.0		
N class					0.363	
N0–N1	27	26.0	77	74.0		
N2–N3	6	18.2	27	81.8		
Stage					0.227	
I–II	25	27.2	67	72.8		
III–IV	8	17.8	37	82.2		
Grade of differentiation					1.000	
I	0	0.0	3	100.0		
II	6	24.0	19	76.0		
III	27	24.8	82	75.2		
Tumor site					0.684	
Tonsil	20	24.1	63	75.9		
Base of tongue	10	28.6	25	71.4		
Soft palate	3	21.4	11	78.6		
Posterior wall of oropharynx	0	0.0	5	100.0		
HPV status					0.265	
HPV +	27	26.5	75	73.5		
HPV −	6	17.1	29	82.9		
CD82 in tumor					**0.029**	
0–1	30	28.8	74	71.2		
2–3	3	9.7	28	90.3		

*HPV* human papillomavirus, *liprin-α1 0–1* negative-weak positivity, *liprin-α1 2–3* moderate-strong positivity. *p* < 0.05

The majority of the patients (77.4%) among the whole cohort presented negative or weak CD82 expression in tumor cells. We saw no correlation between CD82 expression and clinical characteristics (Table 3).

**Table 3:** Clinical characteristics according to CD82 expression in tumor cells

	CD82 0–1	%	CD82 2–3	%	*p* value	Missing / % (*N* = 137)
Number of patients	106	77.4	31	22.6		
Mean age at diagnosis	62.1		60.1		0.296	
Gender					0.280	
Male	79	75.2	26	24.8		
Female	27	84.4	5	15.6		
Smoking habit					0.478	
Non	32	80.0	8	20.0		
Ex	29	70.7	12	29.3		
Current	45	80.4	11	19.6		
Heavy alcohol use					1.000	23 / 16.8
Non	51	77.3	15	22.7		
Ex	12	80.0	3	20.0		
Current	26	78.8	7	21.2		
T class					0.326	
T1–T2	65	74.7	22	25.3		
T3–T4	41	82.0	9	18.0		
N class					0.484	
N0–N1 79	79	76.0	25	24.0		
N2–N3	27	81.8	6	18.2		
Stage					0.722	
I–II	72	78.3	20	21.7		
III–IV	34	75.6	11	24.4		
Grade of differentiation					0.177	
I	1	33.3	2	66.7		
II	19	76.0	6	24.0		
III	86	78.9	23	21.1		
Tumor site					0.811	
Tonsil	63	75.9	20	24.1		
Base of tongue	29	82.9	6	17.1		
Soft palate	10	71.4	4	28.6		
Posterior wall of oropharynx	4	80.0	1	20.0		
HPV status					0.537	
HPV +	81	78.6	22	21.4		
HPV −	25	73.5	9	26.5		

*HPV* human papillomavirus, *CD82 0–1* negative-weak positivity, *CD82 2–3* moderate-strong positivity

### Survival

**Fig. 4:**
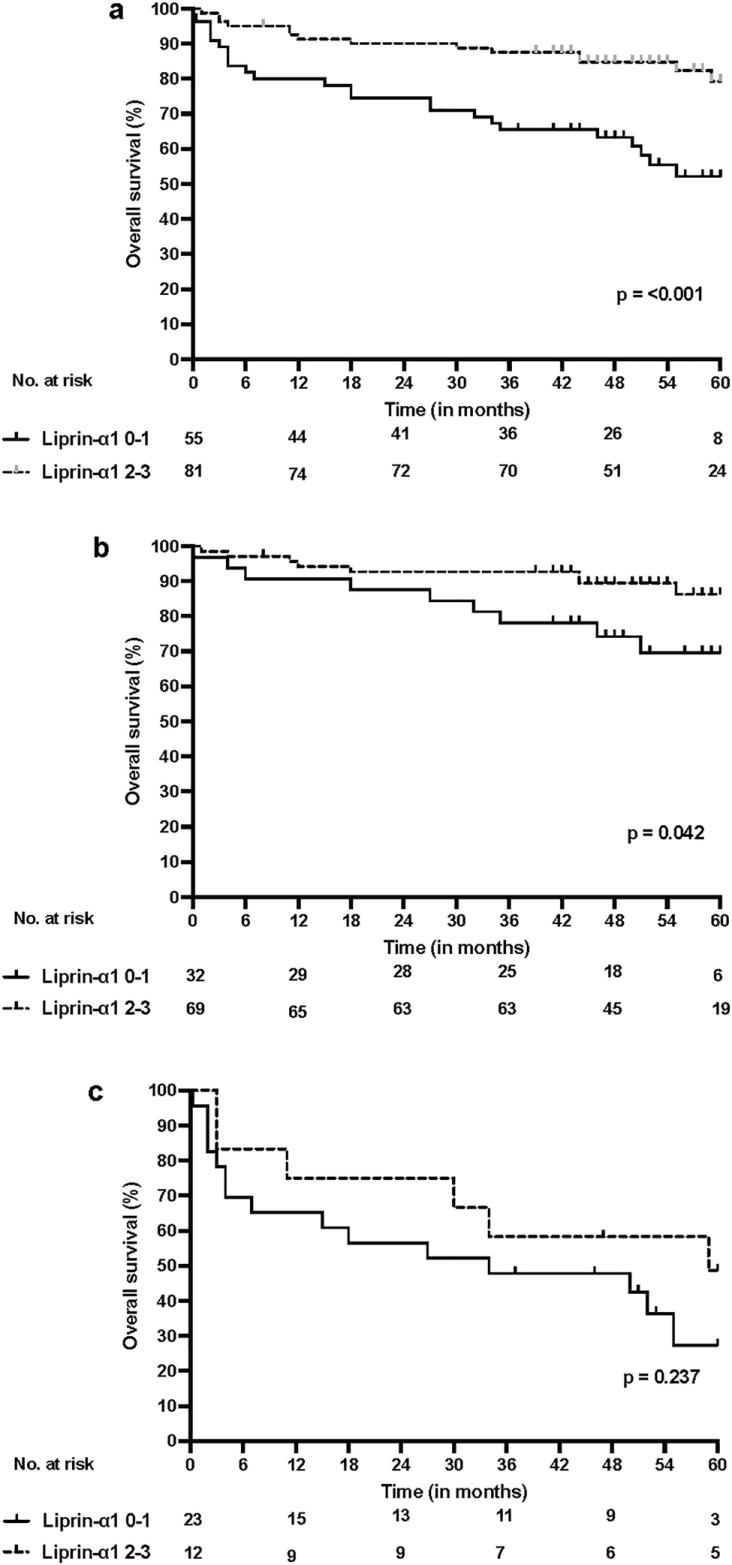
(**a**) Overall survival (OS) according to liprin-α1 expression in TILs in the whole patient cohort (**b**) OS according to liprin-α1 in TILs in HPV-positive patients (**c**) OS according to liprin-α1 expression in TILs in HPV-negative patients

The median follow-up time was 50 months (range 0–60). In the Log-rank analysis, moderate or strong expression of liprin-α1 in TILs correlated with favorable OS among the whole cohort (*p* < 0.001) and among HPV-positive patients (*p* = 0.042). There was no significant correlation in the survival analysis among the HPV-negative subgroup. The Kaplan–Meier curves of the association of OS and liprin-α1 are illustrated in Fig. 4.

In the Cox regression univariable analysis, moderate or strong expression of liprin-α1 in TILs correlated with favorable OS (*p* = 0.002). However, in multivariable analysis, we saw no independent prognostic significance with liprin-α1 expression in TILs (Supplemental Table 1).

There were no associations with survival and elevated expression of liprin-α1 or CD82 in tumor cells.

### HPV Subgroups

Among the entire patient cohort, we found significant correlation between moderate or strong liprin-α1 expressions in TILs and HPV positivity (*p* < 0.001); a majority (68.3%) of the HPV-positive samples presented moderate or strong liprin-α1 expression in TILs (Table 1). Following division according to HPV status, 104 patients (74.8%) altogether were in the HPV-positive subgroup, and 35 patients (25.2%) were in the HPV-negative subgroup.

In the analyses within the subgroups, there was a moderate correlation between male gender and strong liprin-α1 expression in TILs (*p* = 0.049) among the HPV-positive patients. Further, among HPV-positive patients, we found an association with strong liprin-α1 expression and concurrent negative or weak CD82 expression in tumor cells (*p* = 0.006). Among HPV-negative patients, there was a moderate correlation between current smoking habit and negative or weak CD82 expression in tumor cells (*p* = 0.048). The findings according to the HPV subgroup are presented in Supplemental Tables 2 and 3

## Discussion

Our study reports novel findings regarding liprin-α1 expression in OPSCC. The previous findings of Pehkonen et al. have shown the prognostic significance of liprin-α1 in head and neck squamous cell carcinomas [23], and these findings provided the background for our study. Although in our analysis, the majority of the patient samples showed moderate or strong liprin-α1 positivity in tumor cells, no statistically significant association with impaired prognosis was observed. Instead, elevated liprin-α1 expression in the TILs had a favorable association with prognosis, especially in HPV-positive OPSCC patients, but also among the entire patient cohort. Furthermore, we found that elevated liprin-α1 expression in tumor cells correlated with weak tumor expression of CD82, which is in line with a previous finding [16]. Our findings, especially considering liprin-α1 expression in TILs, may be valuable in the future, particularly when considering potential candidates for immunotherapies in both HPV-positive and HPV-negative OPSCC [24].

It is well established that chromosomal region 11q13 is often overexpressed in oral carcinomas [25]. The PPFIA1 gene residing at the 11q13 region encodes liprin-α1 protein, and therefore, we assume that the elevated expression of liprin-α1 in OPSCC could be partly explained by 11q13 amplification. Remarkably, it has recently been discovered that PPFIA1 gene is targeted by miR-142-3p, and possibly downregulated in HPV-positive OPSCC [26]. Additionally, it is possible that the previously observed tumor-promoting functions of liprin-α1 are context-dependent, as has earlier been suggested for breast cancer [15]. Interestingly, Ramos-Garcia et al. additionally suggested that cigarette smoking may associate with the 11q13 amplification, and that HPV has little to no effect on the phenomenon [25]. However, this hypothesis cannot be validated based on our analysis. Further research is needed to define the possible interactions of HPV and liprin-α1 in OPSCC, particularly considering OPSCC’s distinct disease profile compared to other malignancies of the head and neck [4].

Few significant differences were observed between HPV-positive and HPV-negative OPSCC patient subgroups in our analysis. In the HPV-positive subgroup, strong liprin-α1 expression in TILs was associated with favorable OS and male gender. Furthermore, strong liprin-α1 expression in tumor tissue correlated with weak CD82 staining. Interestingly, these results were in line with those of the entire patient cohort. In HPV-negative patients, weak CD82 expression appeared to correlate with current smoking habit. However, it is difficult to assess the impact of this finding, as most of the HPV-negative patients were smokers. Indeed, previous studies on other malignancies have not been able to establish a link between smoking and CD82 regulation [27]. Although our small sample size may limit definitive conclusions, it seems that there are distinct dissimilarities in the clinicopathological characteristics of the expression of liprin-α1 and CD82 between HPV-positive and HPV-negative OPSCC.

According to the present results, strong expression of liprin-α1 in TILs correlated significantly with improved survival among the entire cohort as well as among HPV-positive patients. Remarkably, as mentioned earlier, previous studies have associated liprin-α1 with impaired prognosis in other malignancies when found specifically in tumor cells [16, 17, 28], and therefore, our study presents novel information regarding the possible role of liprin-α1 in the tumor microenvironment (TME), of which TILs are significant components in OPSCC [24]. Although the small number of events in our data may limit robust conclusions on independent prognostic significance, we suggest that elevated liprin-α1 expression in TME is not as detrimental to survival as opposed to elevated expression in the tumor cells, particularly in HPV-positive OPSCC.

As we have previously shown, HPV-positive OPSCC patients often carry a primary tumor with smaller volume compared to patients with HPV-negative OPSCC [4]. Furthermore, the prognostic role of TILs has been thoroughly studied in other malignancies [29–31], and particularly in OPSCC, a high number of TILs have been associated with favorable prognosis and lower T class [20]. Therefore, based on our results, we suggest that liprin-α1 in TME could be one of the factors contributing to the phenomena described above, possibly by restricting tumor growth. We believe that single-cell assays could provide adequate means to assess the functions of liprin-α1 in TME for further research. It is widely recognized that spatial analysis of the expressed proteins and tumor tissues can provide more detailed information on the pathophysiological functions of different biomarkers and their interactions in oncogenic processes [9].

In other malignancies such as breast cancer, it has been shown that liprin has several cancer-promoting functions when found in tumor cells [12, 23, 28], for example, the ability to increase cell motility and extracellular matrix degradation, and thus, invasiveness. However, the functions of liprin-α1 may differ depending on the type of cancer, partly due the complexity and heterogeneity of the 11q13 chromosome region  [13, 25]. Remarkably, it has been suggested that liprin-α1 has invasion-inhibiting abilities in head and neck squamous cell carcinomas [23]. Furthermore, evidence of interactions with a tumor suppressor ING4 in vitro has been found [32]. These phenomena could partly contribute to the favorable prognosis in OPSCC, as is presented in our analysis. Interestingly, in earlier findings with breast cancer by Chiaretti et al., liprin-α1 was found to interact with liprin-β proteins, and liprin-β2 was seen to disrupt cancer cell invasion [14]. However, liprin-β proteins were not observed in our analysis, and thus, their role in OPSCC remains unclear.

In addition to the association with favorable OS, in our analysis, the expression of liprin-α1 in TILs appeared to be linked with lower N class and tumor stage among the entire cohort, and these two clinical characteristics are known to associate with favorable prognosis in OPSCC [33]. Further, strong liprin-α1 expression in TILs was further associated with higher grade, but the impact of this finding is unclear, as the prognostic significance of the grade of differentiation of the tumor is disputable in OPSCC [34].

Although the role of liprin-α1 has previously been studied in head and neck squamous cell carcinomas in vitro [16], to our knowledge, the current analysis is the first clinical study to consider squamous cell carcinomas as well as the association of TILs exclusive to the region of oropharynx, and further involving the HPV status in the analysis. In addition, the strengths of our analysis included extensive follow-up time. The limitations of our study include the limited number of samples.

## Conclusions

Our findings suggest that elevated expression of liprin-α1 in TILs may play a prognostic role in OPSCC and particularly in the HPV-positive disease. However, the details of the pathogenesis were not addressed in the present study. Furthermore, it is possible that in OPSCC, HPV infection may affect liprin-α1 expression and function in tumor cells. Our results, along with several previous intriguing findings, are worthy of more detailed inspection. We are advocating the incorporation of HPV-differentiation analyses in biomarker studies regarding OPSCC to gain more specific information on the role of HPV in the pathogenesis as well as to facilitate treatment individualization. Further research with larger sample sizes and added focus on the pathogenetic aspects are warranted to define the role of liprin-α1 in HPV-positive and HPV-negative OPSCC.

### Supplementary Information

Below is the link to the electronic supplementary material.

Supplementary file1 Table 1: Cox regression multivariable analysis of overall survival (DOCX 16 KB)

Supplementary file2 Table 2: Clinicopathological characteristics according to liprin-α1 and CD82 expression in HPV-positive patients (DOCX 27 KB)

Supplementary file3 Table 3: Clinicopathological characteristics according to liprin-α1 and CD82 expression in HPV-negative patients (DOCX 27 KB)

## Data Availability

Data presented in this study are available on request from the corresponding author. The data are not publicly available due to patient data security.

## Code Availability

Not applicable in this study.
