# Supplementary material for: Liprin-α1 Expression in Tumor-Infiltrating Lymphocytes Associates with Improved Survival in Patients with HPV-Positive Oropharyngeal Squamous Cell Carcinoma
Source: Head Neck Pathol. 2023 Jun 19;17(3):647–57. doi: 10.1007/s12105-023-01565-7 (PMC10513983; doi:10.1007/s12105-023-01565-7)
Supplement: Supplementary file 1 — Supplementary file1 Table 1: Cox regression multivariable analysis of overall survival (DOCX 16 KB) [file 12105_2023_1565_MOESM1_ESM.docx]

Supplemental Table 1 Cox regression multivariable analysis of overall survival

| **Variable** | **OS** | | |
| --- | --- | --- | --- |
|  | **HR** | **95% CI** | ***p*-value** |
| **Age at diagnosis** | 1.054 | 1.012 – 1.097 | **0.011** |
| **Smoking habit** |  |  | 0.152 |
| **Ex versus non-smoker** | 0.807 | 0.271 – 2.766 | 0.807 |
| **Current versus non-smoker** | 2.110 | 0.819 – 5.432 | 0.122 |
| **Stage III-IV versus Stage I-II** | 1.598 | 0.764 – 3.340 | 0.213 |
| **HPV- versus HPV+** | 1.969 | 0.880 – 4.410 | 0.099 |
| **Liprin-α1 0 - 1 versus**  **liprin-α1 2 - 3 in TILs** | 1.520 | 0.714 – 3.240 | 0.278 |

Abbreviations: HR: Hazard ratio; CI: Confidence interval; 0-1: negative/weak expression; 2-3: moderate/strong expression; TILs: Tumor-infiltrating lymphocytes. ***p* < 0.05**
