# Supplementary material for: Liprin-α1 Expression in Tumor-Infiltrating Lymphocytes Associates with Improved Survival in Patients with HPV-Positive Oropharyngeal Squamous Cell Carcinoma
Source: Head Neck Pathol. 2023 Jun 19;17(3):647–57. doi: 10.1007/s12105-023-01565-7 (PMC10513983; doi:10.1007/s12105-023-01565-7)
Supplement: Supplementary file 2 — Supplementary file2 Table 2: Clinicopathological characteristics according to liprin-α1 and CD82 expression in HPV-positive patients (DOCX 27 KB) [file 12105_2023_1565_MOESM2_ESM.docx]

| **Immunostaining** | **liprin-α1 in tumor**  **0 – 1 (%)** | **liprin-α1 in tumor**  **2 - 3 (%)** | ***p*-value** | **liprin-α1 in TILs**  **0 – 1 (%)** | **liprin-α1 in TILs**  **2 - 3 (%)** | ***p*-value** | **CD82 in tumor**  **0 – 1 (%)** | **CD82 in tumor**  **2 – 3 (%)** | ***p*-value** |
| --- | --- | --- | --- | --- | --- | --- | --- | --- | --- |
| **Number of patients** | 27 (26.5) | 75 (73.5) |  | 32 (31.7) | 69 (68.3) |  | 81 (78.6) | 22 (21.4) |  |
| **Mean age at diagnosis** | 61.0 | 60.7 | 0.908 | 60.7 | 60.7 | 0.985 | 61.5 | 58.3 | 0.367 |
| **Gender** |  |  | 0.868 |  |  | **0.049** |  |  | 0.393 |
| Male | 22 (26.8) | 60 (73.2) |  | 22 (27.2) | 59 (72.8) |  | 62 (76.5) | 19 (23.5) |  |
| Female | 5 (25.0) | 15 (75.0) |  | 10 (50.0) | 10 (50.0) |  | 19 (86.4) | 3 (13.6) |  |
| **Smoking habit** |  |  | 0.509 |  |  | 0.291 |  |  | 0.461 |
| Non | 12 (33.3) | 24 (66.7) |  | 12 (34.3) | 23 (65.7) |  | 31 (83.8) | 6 (16.2) |  |
| Ex | 9 (23.1) | 30 (76.9) |  | 9 (23.1) | 30 (76.9) |  | 29 (72.5) | 11 (27.5) |  |
| Current | 6 (22.2) | 21 (77.8) |  | 11 (40.7) | 16 (59.3) |  | 21 (80.8) | 5 (19.2) |  |
| **Heavy alcohol use** |  |  | 0.855 |  |  | 0.156 |  |  | 0.810 |
| Non | 19 (34.5) | 36 (65.5) |  | 15 (27.8) | 39 (72.2) |  | 46 (80.7) | 11 (19.3) |  |
| Ex | 2 (25.0) | 6 (75.0) |  | 5 (62.5) | 3 (37.5) |  | 5 (71.4) | 2 (28.6) |  |
| Current | 5 (31.3) | 11 (68.7) |  | 4 (25.0) | 12 (75.0) |  | 13 (81.3) | 3 (18.7) |  |
| **T class** |  |  | 0.246 |  |  | 0.624 |  |  | 0.079 |
| T1 – T2 | 15 (22.7) | 51 (77.3) |  | 22 (33.3) | 44 (66.7) |  | 49 (73.1) | 18 (26.9) |  |
| T3 – T4 | 12 (33.3) | 24 (66.7) |  | 10 (28.6) | 25 (71.4) |  | 32 (88.9) | 4 (11.1) |  |
| **N class** |  |  | 0.986 |  |  | 0.065 |  |  | 0.757 |
| N0 – N1 | 22 (26.5) | 61 (73.5) |  | 23 (27.7) | 60 (72.3) |  | 65 (77.4) | 19 (22.6) |  |
| N2 – N3 | 5 (26.3) | 14 (73.7) |  | 9 (50.0) | 9 (50.0) |  | 16 (84.2) | 3 (15.8) |  |
| **Stage** |  |  | 0.962 |  |  | 0.116 |  |  | 0.775 |
| I – II | 21 (26.6) | 58 (73.4) |  | 22 (27.8) | 57 (71.2) |  | 62 (77.5) | 18 (22.5) |  |
| III – IV | 6 (26.1) | 17 (73.9) |  | 10 (45.5) | 12 (54.5) |  | 19 (82.6) | 4 (17.4) |  |
| **Grade of differentiation** |  |  | 1.000 |  |  | 0.085 |  |  | 0.062 |
| I | 0 (0.0) | 1 (100.0) |  | 1 (100.0) | 0 (0.0) |  | 0 (0.0) | 1 (100.0) |  |
| II | 2 (28.6) | 5 (71.4) |  | 4 (57.1) | 3 (42.9) |  | 4 (57.1) | 3 (42.9) |  |
| III | 25 (26.6) | 69 (73.4) |  | 27 (29.0) | 66 (71.0) |  | 77 (81.1) | 18 (18.9) |  |
| **Tumor site** |  |  | 0.258 |  |  | 0.091 |  |  | 0.063 |
| Tonsil | 18 (24.7) | 55 (75.3) |  | 20 (27.4) | 53 (72.6) |  | 56 (75.7) | 18 (24.3) |  |
| Base of tongue | 9 (36.0) | 16 (64.0) |  | 9 (37.5) | 15 (62.5) |  | 23 (92.0) | 2 (8.0) |  |
| Soft palate | 0 (0.0) | 4 (100.0) |  | 3 (75.0) | 1 (25.0) |  | 2 (50.0) | 2 (50.0) |  |
| Posterior wall of oropharynx | 0 (0.0) | 0 (0.0) |  | 0 (0.0) | 0 (0.0) |  | 0 (0.0) | 0 (0.0) |  |
| **CD82 in tumor** |  |  | **0.006** |  |  | 0.342 |  |  |  |
| 0 – 1 | 26 (32.9) | 53 (67.1) |  | 26 (33.3) | 52 (66.7) |  |  |  |  |
| 2 – 3 | 1 (4.5) | 21 (95.5) |  | 5 (22.7) | 17 (77.3) |  |  |  |  |
| Abbreviations: HPV: Human papillomavirus, TILs: Tumor-infiltrating lymphocytes, 0–1: negative-weak positivity, 2–3: moderate-strong positivity. ***p* < 0.05** | | | | | |  |  |  |  |

Supplemental Table 2 Clinicopathological characteristics according to liprin-α1 and CD82 expression in HPV-positive patients
