# Supplementary material for: Liprin-α1 Expression in Tumor-Infiltrating Lymphocytes Associates with Improved Survival in Patients with HPV-Positive Oropharyngeal Squamous Cell Carcinoma
Source: Head Neck Pathol. 2023 Jun 19;17(3):647–57. doi: 10.1007/s12105-023-01565-7 (PMC10513983; doi:10.1007/s12105-023-01565-7)
Supplement: Supplementary file 3 — Supplementary file3 Table 3: Clinicopathological characteristics according to liprin-α1 and CD82 expression in HPV-negative patients (DOCX 27 KB) [file 12105_2023_1565_MOESM3_ESM.docx]

| **Immunostaining** | **liprin-α1 in tumor**  **0 -1 (%)** | **liprin-α1 in tumor**  **2 – 3 (%)** | ***p*-value** | **liprin-α1 in TILs**  **0 – 1 (%)** | **liprin-α1 in TILs**  **2 – 3 (%)** | ***p*-value** | **CD28 in tumor**  **0 - 1 (%)** | **CD82 in tumor**  **2 – 3 (%)** | ***p*-value** |
| --- | --- | --- | --- | --- | --- | --- | --- | --- | --- |
| **Number of patients** | 6 (17.1) | 29 (82.9) |  | 23 (65.7) | 12 (34.3) |  | 25 (73.5) | 9 (26.5) |  |
| **Mean age at diagnosis** | 63.2 | 63.9 | 1.000 | 64.9 | 61.7 | 0.313 | 63.9 | 64.7 | 0.814 |
| **Gender** |  |  | 1.000 |  |  | 0.434 |  |  | 0.692 |
| Male | 4 (16.0) | 21 (84.0) |  | 15 (60.0) | 10 (40.0) |  | 17 (70.8) | 7 (29.2) |  |
| Female | 2 (20.0) | 8 (80.0) |  | 8 (80.0) | 2 (20.0) |  | 8 (80.0) | 2 (20.0) |  |
| **Smoking habit** |  |  | 0.634 |  |  | 1.000 |  |  | **0.048** |
| Non | 1 (33.3) | 2 (66.7) |  | 2 (33.3) | 1 (66.7) |  | 1 (33.3) | 2 (66.7) |  |
| Ex | 0 (0) | 2 (100.0) |  | 1 (50.0) | 1 (50.0) |  | 0 (0) | 1 (100.0) |  |
| Current | 5 (16.7) | 25 (83.3) |  | 20 (66.7) | 10 (33.3) |  | 24 (80.0) | 6 (20.0) |  |
| **Heavy alcohol use** |  |  | 1.000 |  |  | 0.306 |  |  | 0.356 |
| Non | 2 (20.0) | 8 (80.0) |  | 8 (80.0) | 2 (20.0) |  | 5 (55.6) | 4 (44.4) |  |
| Ex | 1 (12.5) | 7 (87.5) |  | 6 (75.0) | 2 (25.0) |  | 7 (87.5) | 1 (12.5) |  |
| Current | 3 (17.6) | 14 (82.4) |  | 9 (52.9) | 8 (47.1) |  | 13 (76.5) | 4 (23.5) |  |
| **T class** |  |  | 1.000 |  |  | 0.282 |  |  | 0.435 |
| T1 – T2 | 4 (19.0) | 17 (81.0) |  | 12 (57.1) | 9 (42.9) |  | 16 (80.0) | 4 (20.0) |  |
| T3 – T4 | 2 (14.3) | 12 (85.7) |  | 11 (78.6) | 3 (21.4) |  | 9 (64.3) | 5 (35.7) |  |
| **N class** |  |  | 0.366 |  |  | 0.282 |  |  | 0.704 |
| N0 – N1 | 5 (23.8) | 16 (76.2) |  | 12 (57.1) | 9 (42.9) |  | 14 (60.0) | 6 (40.0) |  |
| N2 – N3 | 1 (7.1) | 13 (92.9) |  | 11 (78.6) | 3 (21.4) |  | 11 (78.6) | 3 (21.4) |  |
| **Stage** |  |  | 0.166 |  |  | 0.079 |  |  | 0.439 |
| I – II | 4 (30.8) | 9 (69.2) |  | 6 (46.2) | 7 (53.8) |  | 10 (83.3) | 2 (16.7) |  |
| III – IV | 2 (9.1) | 20 (90.9) |  | 17 (77.3) | 5 (22.7) |  | 15 (68.2) | 7 (31.8) |  |
| **Grade of dirrentiation** |  |  | 0.771 |  |  | 0.641 |  |  | 0.291 |
| I | 0 (0.0) | 2 (100.0) |  | 1 (50.0) | 1 (50.0) |  | 1 (50.0) | 1 (50.0) |  |
| II | 4 (22.2) | 14 (77.8) |  | 11 (61.0) | 7 (38.9) |  | 15 (83.3) | 3 (16.7) |  |
| III | 2 (13.3) | 13 (86.7) |  | 11 (73.3) | 4 (26.7) |  | 9 (64.3) | 5 (35.7) |  |
| **Tumor site** |  |  | 0.646 |  |  | 1.000 |  |  | 0.790 |
| Tonsil | 2 (20.0) | 8 (80.0) |  | 7 (70.0) | 3 (30.0) |  | 7 (77.8) | 2 (22.2) |  |
| Base of tongue | 1 (10.0) | 9 (90.0) |  | 7 (70.0) | 3 (30.0) |  | 6 (60.0) | 4 (40.0) |  |
| Soft palate | 3 (30.0) | 7 (70.0) |  | 6 (60.0) | 4 (40.0) |  | 8 (80.0) | 2 (20.0) |  |
| Posterior wall of oropharynx | 0 (0.0) | 5 (100.0) |  | 3 (60.0) | 2 (40.0) |  | 4 (80.0) | 1 (20.0) |  |
| **CD82 in tumor** |  |  | 0.644 |  |  | 1.000 |  |  |  |
| 0-1 | 4 (16.0) | 21 (84.0) |  | 17 (68.0) | 8 (32.0) |  |  |  |  |
| 2-3 | 2 (22.2) | 7 (77.8) |  | 6 (66.7) | 3 (33.3) |  |  |  |  |
| Abbreviations: HPV: Human papillomavirus, TILs: Tumor-infiltrating lymphocytes. 0–1: negative-weak positivity, 2–3: moderate-strong positivity.  ***p* < 0.05** | | | | | |  |  |  |  |

Supplemental Table 3 Clinicopathological characteristics according to liprin-α1 and CD82 expression in HPV-negative patients
